# Real world experience on the effectiveness and safety of pirfenidone in patients with idiopathic pulmonary fibrosis in Taiwan

**DOI:** 10.3389/fmed.2023.1242260

**Published:** 2023-10-30

**Authors:** Cheng-Yu Chang, Yu-Feng Wei, Chung-Yu Chen, Yi-Chun Lai, Po-Wei Hu, Jui-Chi Hung, Chi-Hsiang Chu, Hsin-Tzu Chuang, Shih-Chieh Chang

**Affiliations:** ^1^Division of Chest Medicine, Department of Internal Medicine, Far Eastern Memorial Hospital, New Taipei, Taiwan; ^2^Department of Nursing, Cardinal Tien Junior College of Healthcare and Management, New Taipei, Taiwan; ^3^School of Medicine for International Students, College of Medicine, I-Shou University, Kaohsiung, Taiwan; ^4^Department of Internal Medicine, E-Da Cancer Hospital, I-Shou University, Kaohsiung, Taiwan; ^5^Department of Internal Medicine, National Taiwan University Hospital Yunlin Branch, Yunlin, Taiwan; ^6^College of Medicine, National Taiwan University, Taipei, Taiwan; ^7^Division of Chest Medicine, Department of Internal Medicine, National Yang-Ming Chiao Tung University Hospital, Yilan, Taiwan; ^8^Faculty of Medicine, College of Medicine, National Yang-Ming Chiao Tung University, Taipei, Taiwan; ^9^Department of Statistics, Tunghai University, Taichung, Taiwan; ^10^School of Pharmacy, Kaohsiung Medical University, Kaohsiung, Taiwan; ^11^Department of Critical Care Medicine, National Yang-Ming Chiao Tung University Hospital, Yilan, Taiwan

**Keywords:** pirfenidone, idiopathic pulmonary fibrosis, tumor necrosis factor alpha, progression free survival, forced vital capacity

## Abstract

**Introduction:**

Randomized controlled trials have demonstrated a reduction in the decline of lung function and a reduced risk of acute exacerbation in patients with idiopathic pulmonary fibrosis treated with the antifibrotic prifenidone. The present study aimed to investigate the real-world effectiveness and safety profile of pirfenidone treatment for patients with IPF in Taiwan.

**Methods:**

Between January 1, 2019 and December 31, 2020, we enrolled 50 patients who were newly diagnosed with IPF and had at least 12 months follow-up period after pirfenidone administration.

**Result:**

The primary outcome of pharmacologic effect showed that the mean differences in the absolute values of forced vital capacity from baseline were 0.2 liter (*n* = 36), 0.13 liter (*n* = 32), 0.04 liter (*n* = 26), and − 0.004 liter (*n* = 26) after 3, 6, 9, and 12 months of administration, respectively. A slight improvement in quality of life, including scores of chronic obstructive pulmonary disease assessment test and St. George’s respiratory questionnaire scores. The most common adverse effects were gastrointestinal upset and dermatological problems. No new safety concerns were observed in the present study.

**Conclusion:**

Our real-world study describe for the first time in Taiwan, the use of pirfenidone over a 12 months period. This drug preserves the lung function and improves quality of life with tolerable side effects.

## Introduction

Idiopathic pulmonary fibrosis (IPF) is characterized by chronic and progressive fibrosis of the lung parenchyma due to unknown causes. Patients with IPF experience progressive shortness of breath and decline in pulmonary function (1). Despite its chronicity, acute exacerbation occurs and is an important factor for mortality and disease progression. The prognosis of IPF is poor, with a median survival of 2–5 years (2).

Non-pharmacological management of IPF includes supplemental oxygen therapy, pulmonary rehabilitation, and lung transplantation. Additionally, pirfenidone and nintedanib are the first two antifibrotic agents that have proven to be effective and safe in treating IPF patients (3).

Pirfenidone is a small molecule with anti-fibrotic and anti-inflammatory activities that is administered orally. It targets transforming growth factor beta (TGF-β) and tumor necrosis factor alpha (TNF-ɑ) pathways, leading to decreased collagen synthesis and fibroblast proliferation (4).

Four phase III trials were conducted to evaluate the effectiveness and safety of pirfenidone in patients with IPF. In a Japanese multicenter, double-blinded, placebo-controlled study, pirfenidone treatment was associated with decreased vital capacity (VC) decline (−0.09 L in pirfenidone 1800 mg/day vs. −0.16 L in placebo group) and better progression free survival (PFS) over 52 weeks (5). Pooled data from two CAPACITY (Clinical Studies Assessing Pirfenidone in Idiopathic Pulmonary Fibrosis: Research on Efficacy and Safety Outcomes) trials showed a 22.8% relative reduction in forced vital capacity (FVC) decline and 26% improvement in progression-free survival (pirfenidone 2,403 mg/day vs. placebo) (6). The ASCEND (Assessment of Pirfenidone to Confirm Efficacy and Safety in IPF) study confirmed the results of the CAPACITY study in that there was a relative reduction of 47.9% in patients who had a 10% or more absolute decline in FVC over 52 weeks; six-minute walking distance decline was also reduced in the experimental arm (7).

Pirfenidone was well tolerated, and the most common adverse events were nausea, dyspepsia, vomiting, anorexia, photosensitivity, and dizziness. Although it was more common in the pirfenidone group than in the placebo group, treatment discontinuation was rare (8).

Based on these phase III trials, pirfenidone was approved in Japan in 2008 as the first anti-fibrotic drug for IPF treatment. Later, it was licensed and approved by the European Medicines Agency (EMA) in 2011 and the US Food and Drug Administration (FDA) in 2014 (9). It was not until May 2016 that it was approved by the Taiwan FDA and reimbursed by the national health insurance since 2017.

In addition to randomized controlled trials, treatment outcomes in real-world clinical practice are usually confounded by many factors such as comorbidities, medical adherence, and concurrent medications. In this study, we aimed to investigate the real-world effectiveness and safety profile of pirfenidone treatment in patients with IPF in Taiwan.

## Materials and methods

### Study design

We conducted a multicenter observational trial in one medical center and three teaching hospitals in Taiwan. The institutional review board at each center approved the protocol [National Yang-Ming Chiao Tung University Hospital (IRB No.: 2020D003), Far-Eastern Memorial Hospital (IRB No.: 108096-F), E-DA Hospital (IRB No.: EMRP19108N) and National Taiwan University Hospital Yunlin Branch (IRB No.: 202103132RINB)]. Effectiveness and safety results were reviewed for a total of 24 months, from January 1, 2019, to December 31, 2020. All study participants have signed an informed consent form, and the consent form has also been approved by the IRB institutional for human trial.

### Inclusion and exclusion criteria

The inclusion criteria were (1) patients with IPF using pirfenidone, diagnosed by the latest 2018 ATS/ERS/JRS/ALAT IPF guideline (1), (2) age >20 years, (3) willing to perform pulmonary function tests and questionnaires and provide informed consent, (4) the FVC was approximately 50%–80%.

The exclusion criteria were as follows: (1) life expectancy of <12 months, (2) unable to perform pulmonary function test or complete questionnaires, (3) contraindication to pirfenidone use, (4) pregnancy or preparation for pregnancy, (5) other etiologies of interstitial lung disease including drug use, autoimmune disease, occupational exposure, or infectious disease, (6) ongoing or historical use of methotrexate, amiodarone, nitrofurantoin, rituximab, sulfasalazine, or other treatments related to interstitial lung disease, (7) chronic liver disease, Child-Pugh class C, (8) end-stage renal disease requiring dialysis, (9) breastfeeding women.

### Enrollment and follow-up intervals

IPF was defined according to an international consensus statement (1). All enrolled IPF patients met the National Health Insurance (NHI) pirfenidone criteria of 24 weeks medicine use. FVC measurement was asked to perform every 24 weeks since the start of pirfenidone prescription. In the event that an IPF patient did not meet the NHI pirfenidone application criteria with an FVC decrease of more than 10%, pirfenidone use was stopped. We continued to follow IPF patient data to the end of the study.

Various items and parameters (Table 1) were measured from the start of pirfenidone use, and at the third, sixth, ninth, and 12th month with regular liver, renal function, high resolution chest tomography (HRCT), pulmonary function test, St. George’s respiratory questionnaire (SGRQ), and chronic obstructive pulmonary disease (COPD) assessment test (CAT).

**Table 1:** Follow items from the beginning of pirfenidone use.

Items/months	0	3	6	9	12
AST/ALT, creatinine	V	V	V	V	V
Autoimmune profile	V				
HRCT	V		V		V
Pulmonary function test	V	V	V	V	V
St. George’s respiratory questionnaire	V	V	V	V	V
COPD assessment test	V	V	V	V	V

AST, aspartate aminotransferase; ALT, alanine aminotransferase; COPD, chronic obstructive pulmonary disease; HRCT, high resolution chest tomography.

### End points

The primary endpoint was the change in pulmonary function test results (FVC and diffusing capacity) after pirfenidone use at the third, sixth, ninth and 12th month. Secondary endpoints were the time to disease progression, SGRQ, CAT, mortality rate, drug dosage, and side effects.

### Statistical analysis

SPSS (version 22; IBM Corporation, Armonk, NY, United States) was used for statistical analysis of the clinical data. Data were calculated as frequencies for categorical variables and means (standard deviations) for continuous variables. Categorical variables were compared using the chi-square test or Fisher’s exact test, while continuous variables were compared using the Kruskal–Wallis test or Mann–Whitney *U* test. To deal with missing data, we consider three methods: using complete data and two imputation techniques, last observation carried forward (LOCF) and multiple imputation (MI), for sensitivity analysis. Progression-free survival and overall survival (OS) were assessed using Kaplan–Meier survival curves, and statistical differences were calculated using the log-rank test. Statistical significance was set at *p* < 0.05. Questionnaires including the SGRQ, CAT, and a case report form were attached with supplements. SPSS version 19 (SPSS Inc., Chicago, IL, United States) and R package (R Foundation for Statistical Computing, Vienna, Austria) were used for statistical analysis.

### Ethics statement

The studies involving human participants were reviewed and approved by the Institutional Review Board (IRB) and Ethics Committee of National Yang-Ming Chiao Tung University Hospital (IRB No.: 2020D003), Far-Eastern Memorial Hospital (IRB No.: 108096-F), E-DA Hospital (IRB No.: EMRP19108N) and National Taiwan University Hospital Yunlin Branch (IRB No.: 202103132RINB).

## Results

Between January 1, 2019, and December 31, 2020, 50 patients newly diagnosed with IPF were enrolled in this study with at least 12 months follow-up period after pirfenidone administration. Patient demographic data are summarized (Table 2). A total 38 (76%) patients were male, and the mean age at IPF diagnosis was 74 years. Twenty-six (52%) patients were ex-smokers or current smokers. The most common comorbidity was gastroesophageal reflux disease followed by diabetes mellitus. Baseline pulmonary function tests and respiratory symptom assessment, including the Modified Medical Research Council (mMRC) dyspnea scale, SGRQ, and CAT, were measured before the start of pirfenidone treatment. In general, patients exhibited impaired physiological parameters and quality of life (QOL). The mean values of FVC and diffusing capacity for carbon monoxide (DLCO) (% of the predicted value) were 66.4% and 55.5%, respectively. Concurrent medications included only 1 patient (2%) receiving immunosuppressant (methotrexate) and 8 patients (16%) with prednisolone less than 10 mg/day, and 6 patients (12%) with prednisolone more than 10 mg/day. Nine patients received GERD treatment with esomeprazole (2 patients), H2 blocker (6 patients) and antacid use (1 patient).

**Table 2:** Baseline characteristics (total enrolled population, *n* = 50).

Withdraw in 1 year	5 (10.0%)
Death in 1 year	8 (16.0%)
Male gender	38 (76%)
Age, years	74.1 ± 10.1
Median [min., max.]	74.4 [51.2, 92.8]
Age group ≥65 years	42 (84.0%)
Weight, kg	62.5 ± 12.7
BMI	24.2 ± 4.0
**Cigarette smoking history**	
Present	6 (12.0%)
Former	20 (40.0%)
Never	24 (48.0%)
Time since diagnosis, years	0.51 ± 0.90
Median [min., max.]	0.14 [0, 5.03]
**Comorbidities**	
Gastroesophageal reflux (GER)	9 (18.0%)
GER Treatment (% under GER)	9 (100.0%)
Diabetes mellitus	5 (10.0%)
Familial pulmonary fibrosis	1 (2%)
Cancer history	6 (12.0%)
Dyspnea (MMRC)	1.76 ± 0.73
MMRC = 0, 1	20 (40.0%)
MMRC = 2	22 (44.0%)
MMRC = 3, 4	8 (16.0%)
**Spirometry**	
DLCO (% predicted) (*n* = 30)	55.49 ± 20.62
FVC (% predicted)	66.37 ± 11.36
>80%	6 (12.0%)
60%–80%	29 (58.0%)
50%–60%	13 (26.0%)
≤50%	2 (4.0%)
FVC (L)	1.84 ± 0.56
SGRQ (*n* = 38)	43.81 ± 20.85
Median [min., max.]	46.10 [1.05, 83.8]
COPD Assessment Test (CAT) (*n* = 43)	17.13 ± 6.42
Median [min., max.]	16 [3, 35]
**Concurrent medications**	
Methotrexate	1 (2.0%)
*Prednisolone*	
Daily dose <10 mg	8 (16.0%)
Daily dose ≥10 mg	6 (12.0%)
*GERD medication*	
H2 blocker	6 (12.0%)
Proton-pump inhibitor	2 (4.0%)
Antacid	1 (2.0%)

Data are presented as *n* (%), mean ± SD, or as indicated. BMI, body mass index; MMRC, Modified Medical Research Council; DLCO, diffusing capacity of the lung for carbon monoxide; FVC, forced vital capacity; SGRQ, St. George’s respiratory questionnaire; COPD, chronic obstructive pulmonary disease.

**Figure 1:**
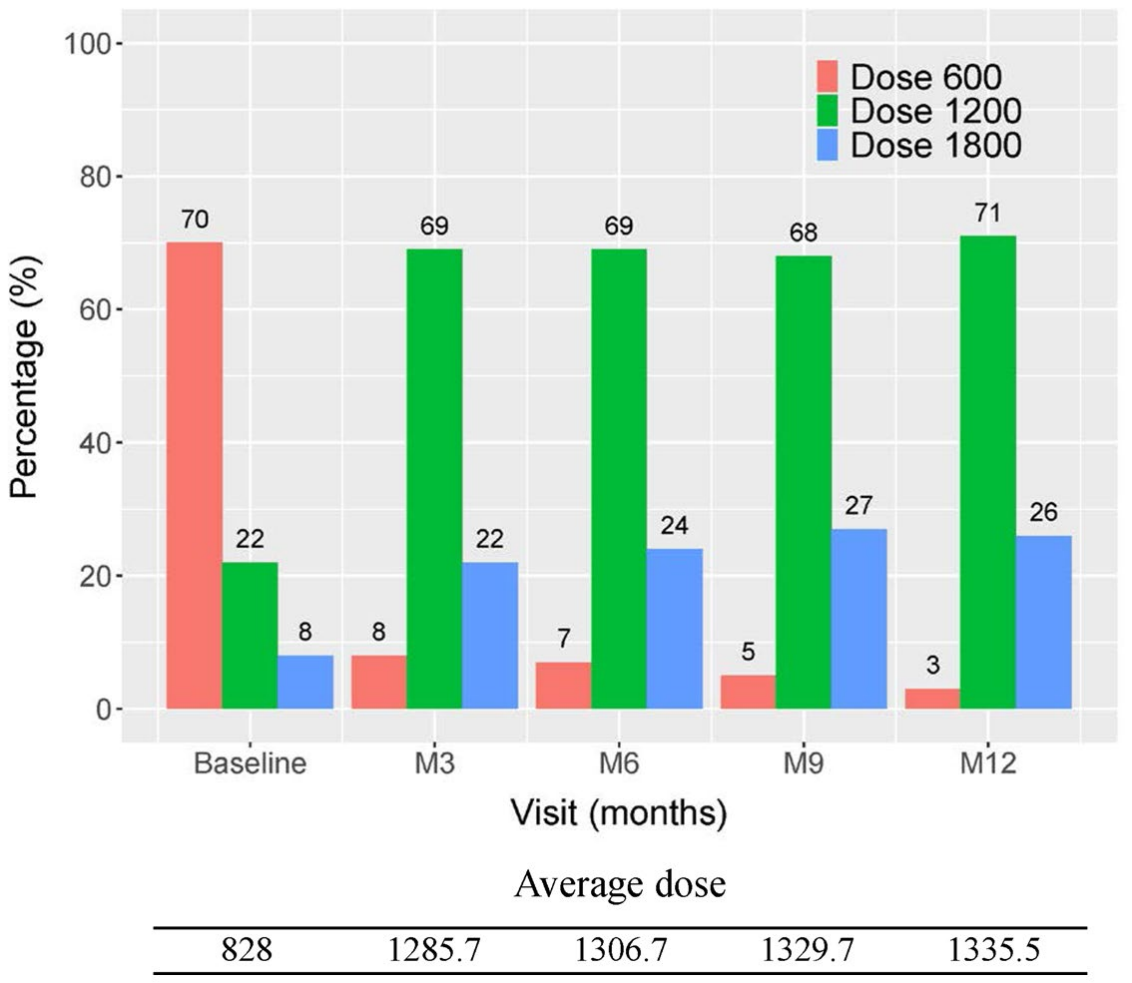
The average usage dose of pirfenidone in every 3 months visit.

**Figure 2:**
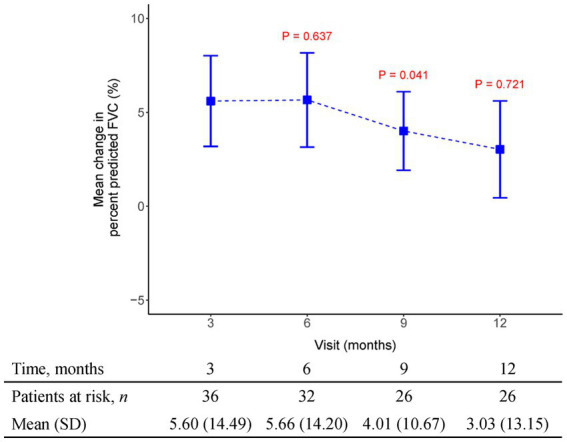
The mean change from baseline value in percent predicted FVC in every 3 months visit. *p*-value is calculated by the comparison between the time points M12 and M3.

**Figure 3:**
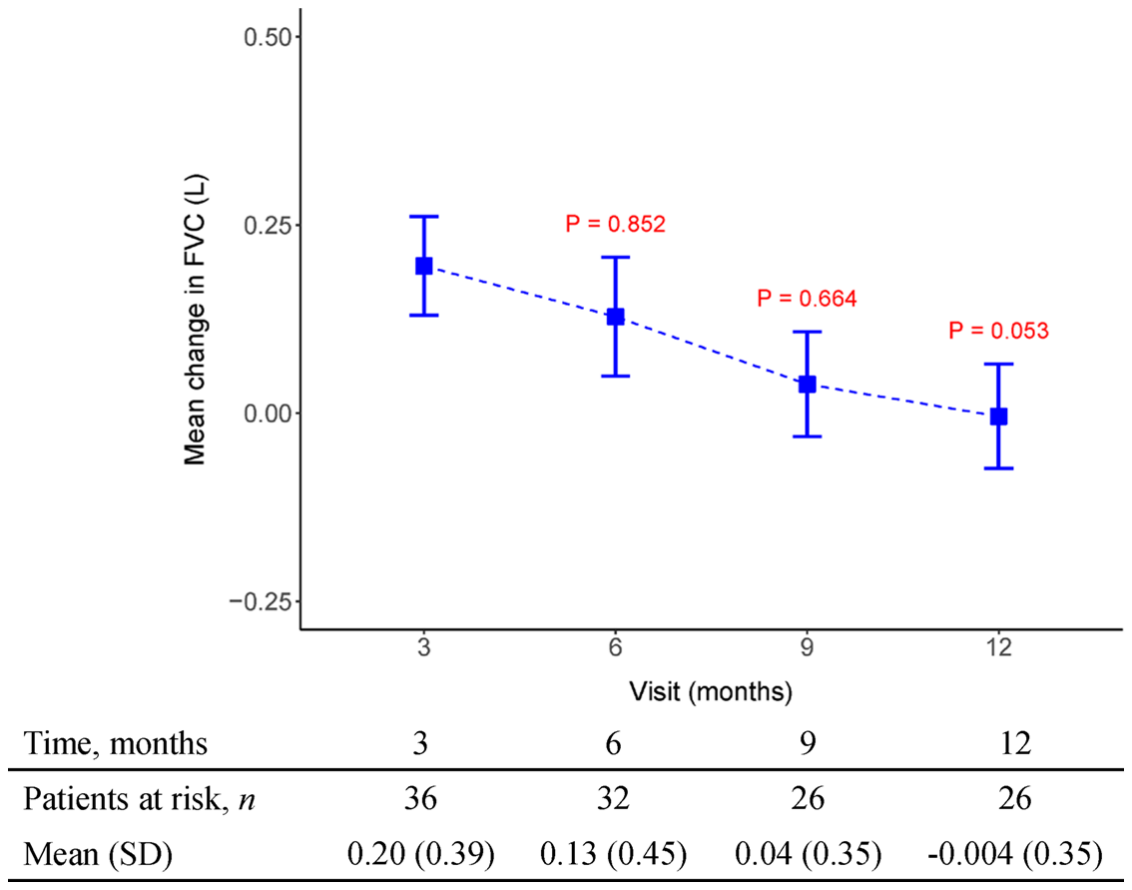
The mean change from baseline value in FVC (L) in every 3 months visit. *p*-value is calculated by the comparison between the time points M12 and M3.

**Figure 4:**
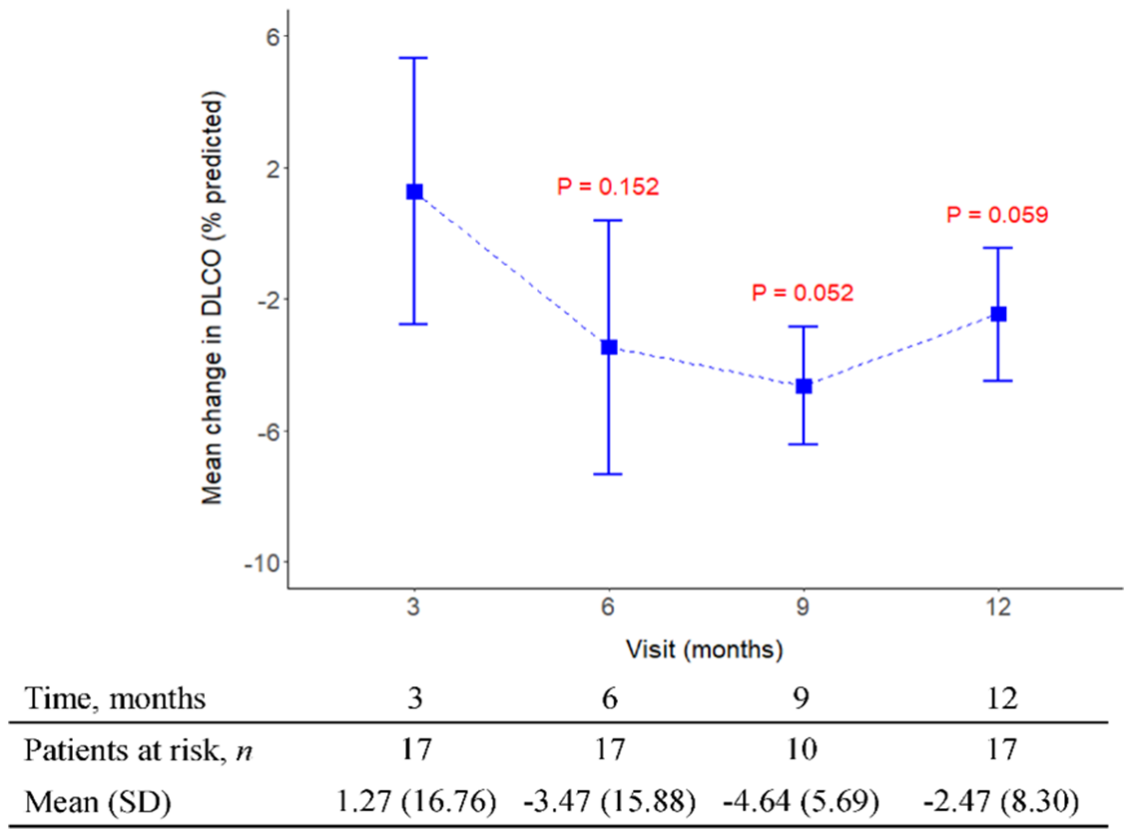
The mean change from baseline value in DLCO (%) in every 3 months visit. *p*-value is calculated by the comparison between the time points M12 and M3.

**Figure 5:**
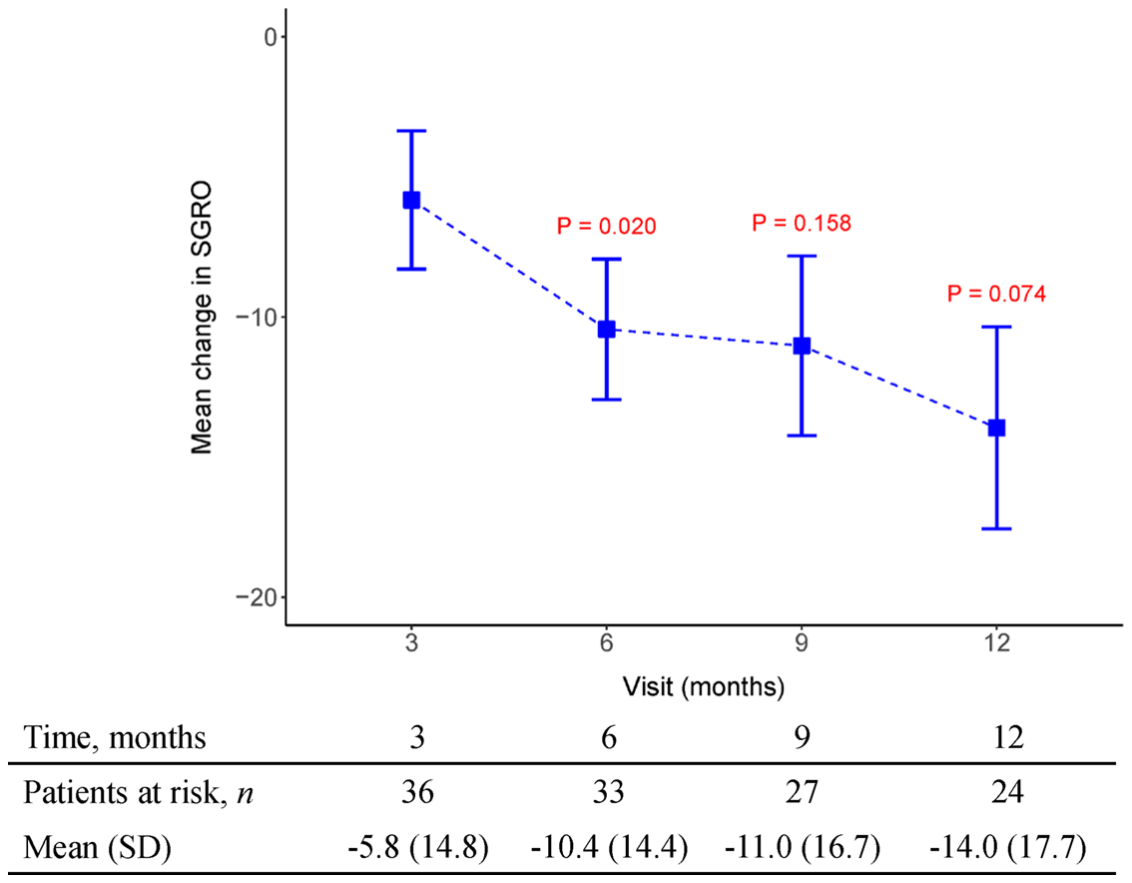
The mean change from baseline value in SGRQ in every 3 months visit. *p*-value is calculated by the comparison between the time points M12 and M3.

**Figure 6:**
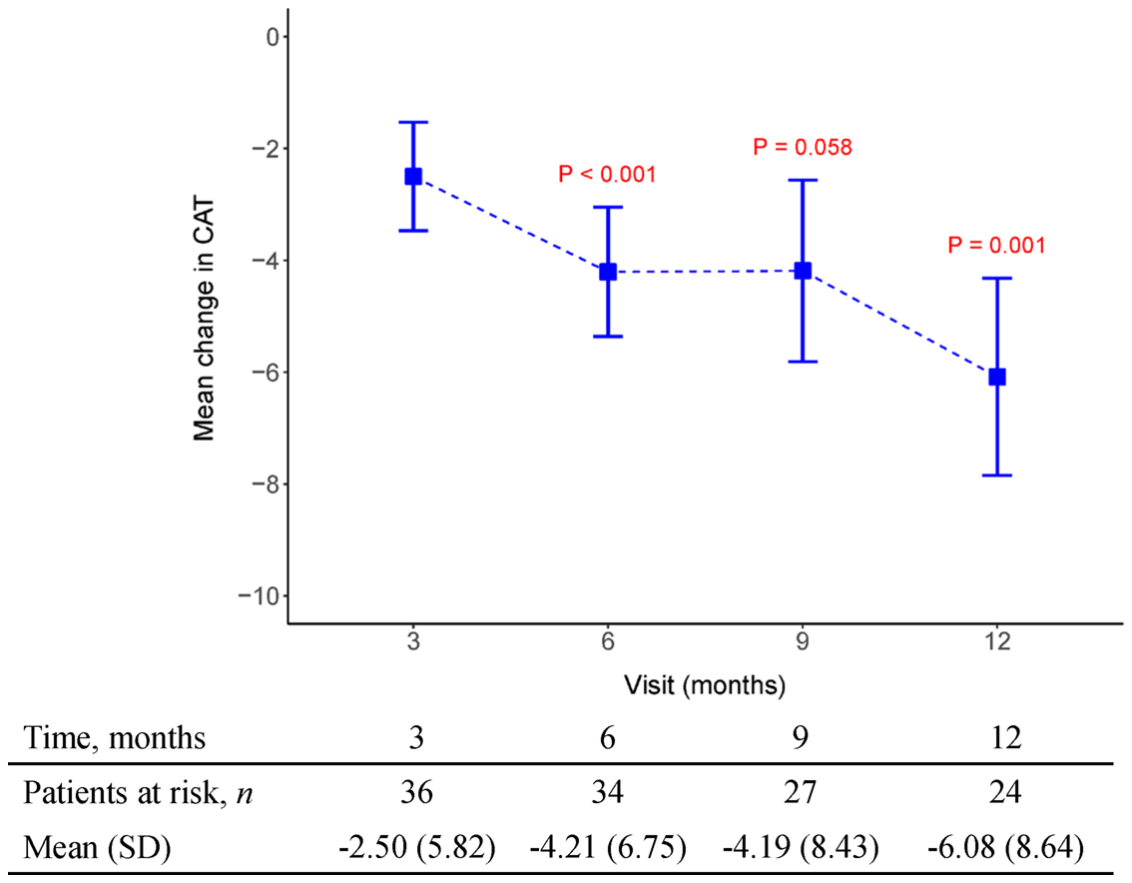
The mean change from baseline value in CAT in every 3 months visit. *p*-value is calculated by the comparison between the time points M12 and M3.

The initial pirfenidone dosage was 600 mg daily and escalated according to the patient’s tolerance. At 12 months, 71%, 26%, and 3% of patients received 1,200 mg, 1800 mg daily, and 600 mg daily doses of pirfenidone, respectively (Figure 1). The primary outcome of pharmacologic effect (Figures 2, 3) showed that the mean differences in FVC absolute value (% of predicted value) from baseline were 0.2 liter (5.6%, *n* = 36), 0.13 liter (5.7%, *n* = 32), 0.04 liter (4%, *n* = 26), and −0.004 liter (3%, *n* = 26) at 3, 6, 9, and 12 months, respectively. Although there was quite a few missing data of DLCO, there was no statistically significant difference comparison to the baseline value (Figure 4). A slight improvement in quality of life, including CAT and SGRQ scores, also demonstrated clinically significant improvement in patients remaining on pirfenidone treatment (Figures 5, 6).

Supplementary Figures S1–S5 depict changes from baseline to month 12 using three different methods for handling missing data. There is no statistically significant difference in predicted FVC (%) over time (see Supplementary Table S1). In FVC (L), there is also no statistically significant difference under using complete data and LOCF imputation. However, multiple imputation shows a significant difference at M3 and M6, but it also indicates that there is no statistically significant difference between baseline and M12 (see Supplementary Table S2). Supplementary Tables S3–S5 demonstrate a significant difference over time in predicted DLCO (%), SGRQ, and CAT, especially indicating a statistically significant difference between baseline and month 12. Questionnaires including the SGRQ, CAT, and a case report form were attached with supplements.

**Figure 7:**
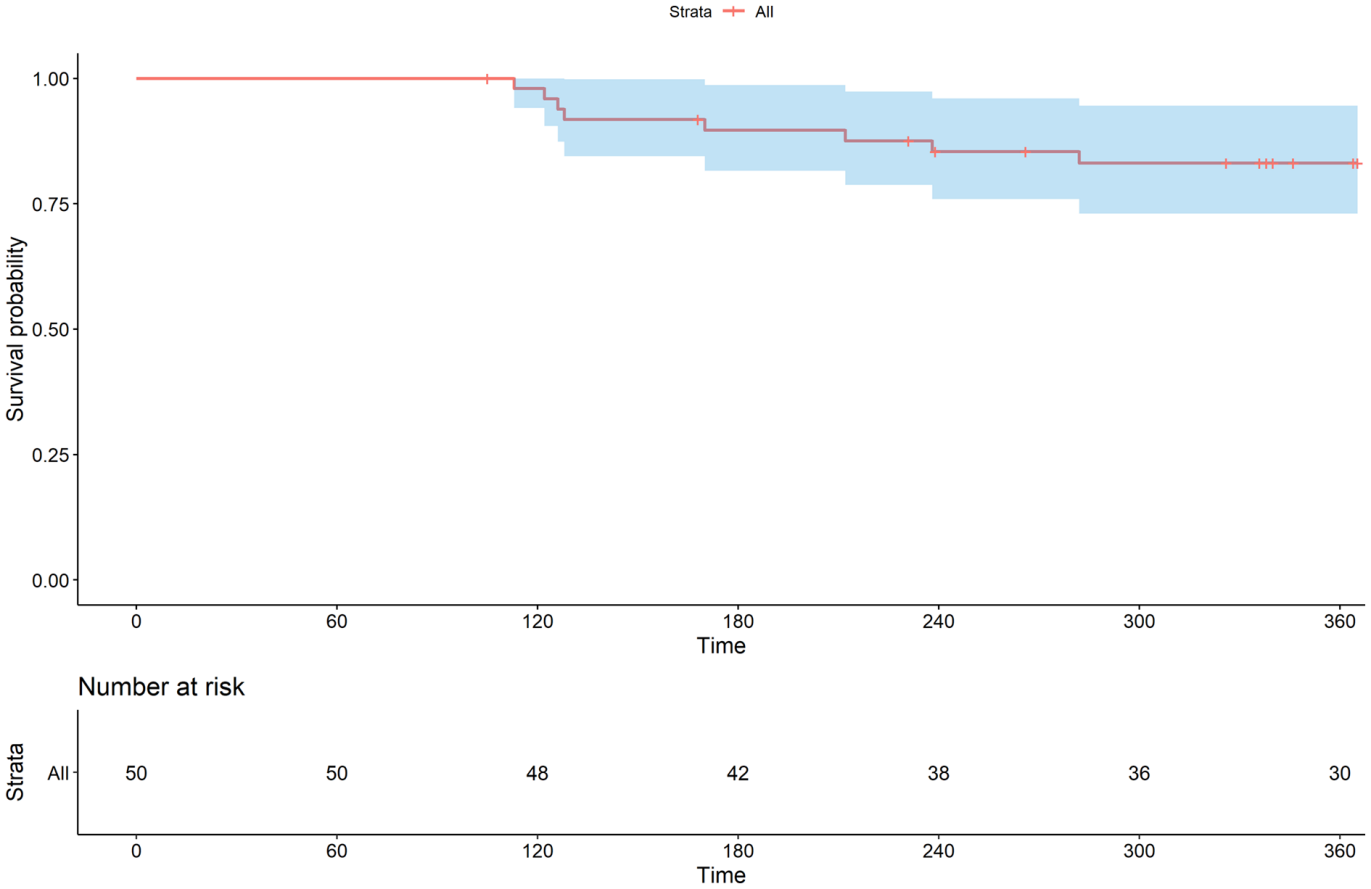
Kaplan–Meier curve for overall survival time.

**Figure 8:**
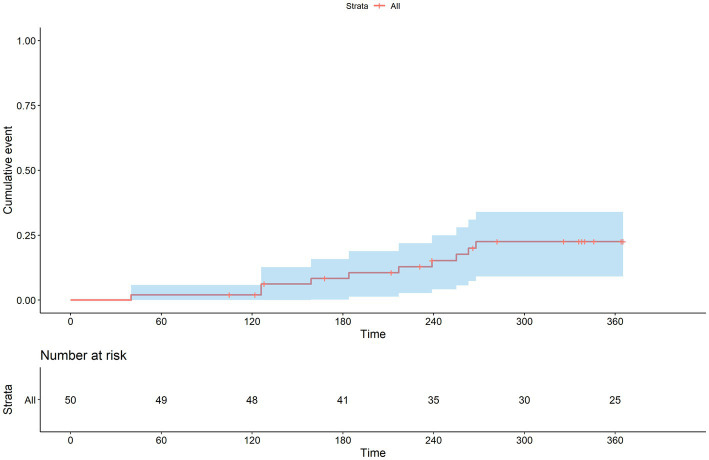
Kaplan–Meier curve for time to first acute exacerbation.

The Kaplan–Meier curves for all-cause mortality and acute exacerbation during the study period are shown in Figures 7, 8. The mean time to acute exacerbation was 188 ± 75 days. An absolute decline of more than 10% in FVC occurred in nine (18%) patients at the 12 months follow-up.

**Figure 9:**
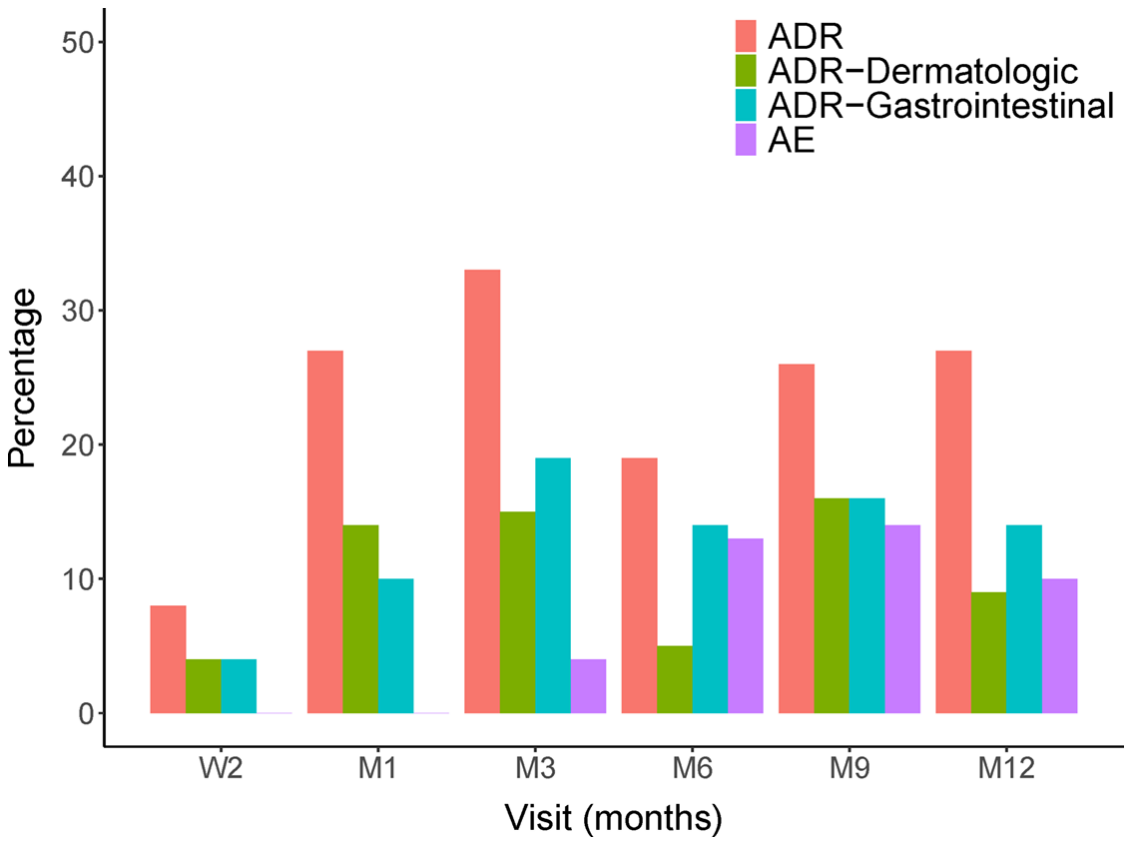
The adverse drug reaction in every 3 months visit.

No new safety concerns were observed in the present study. The most common adverse effects were gastrointestinal upset and dermatological problems, such as photosensitivity, which were usually ameliorated with symptomatic treatment or dose adjustment. Side effects were most likely to occur after pirfenidone intake for 3 months (Figure 9). Only two (4%) patients discontinued the drug due to adverse events, and five (10%) patients underwent dose adjustment due to adverse events. The overall all-cause mortality rate was 16% (Table 3).

**Table 3:** Acute exacerbation, death and adverse drug reaction (total enrolled population, *n* = 50).

Variable	Events, *n*	Patients, *n* (%)
Acute exacerbation (AE)	—	10 (20.0%)
Count = 0		40 (80.0%)
Count = 1		7 (14.0%)
Count ≥2		3 (6.0%)
Time to first AE (day, mean ± SD)	188 ± 75	
Death	—	8 (16.0%)
Adverse drug reaction (pirfenidone related)	110	26 (52.0%)
Discontinuation due to adverse events		2 (4%)
Dose adjustment due to adverse events		5 (10%)
ADR-dermatologic	35	14 (28.0%)
ADR-gastrointestinal	60	16 (32.0%)
ADR-cardiovascular	0	0 (0.0%)
ADR-psychoneurologic	4	3 (6.0%)
ADR-hepatic	4	3 (5.2%)
ADR-hematologic	0	0 (0.0%)
ADR-others	7	4 (8.0%)

## Discussion

Pirfenidone received approval from the Taiwan Food and Drug Administration in 2016 and the reimbursement criteria are restricted to patients with a confirmed diagnosis of idiopathic pulmonary fibrosis (IPF) or a definite usual interstitial pneumonia pattern observed on high-resolution CT scans. According to the strict payment rule, we included patients with lung function in the FVC range of 50% to 80%, and at least two pulmonary specialists reviewed the HRCT images. This pilot observational real-world study in Taiwan showed that 50 patients treated with pirfenidone were enrolled in the NHI payment group at any time during enrollment and follow-up period (January 1, 2019, to December 31, 2020). According to evidence-based guidelines for the management of IPF, it has been established that a decline in FVC serves as a predictive factor for mortality in patients diagnosed with IPF (10). While a decline in vital capacity (VC) or FVC greater than 10% is commonly considered an indicative marker of disease progression in patients with IPF, recent studies suggest that even a marginal decline of 5% to 10% in FVC holds clinical significance for prognosis. Previous reports have highlighted the importance of monitoring even subtle changes in FVC as they may provide valuable insights into the patient’s overall prognosis (11). In an extended analysis of a phase III clinical trial evaluating the efficacy of pirfenidone in the treatment of IPF, another study revealed that a 5% alteration in VC measured 3 months after initiating pirfenidone therapy could serve as a predictive factor for clinical efficacy over a 12 months period. This finding underscores the potential value of early VC assessment as an indicator of treatment response and long-term outcomes in IPF patients undergoing pirfenidone treatment (12). In our study, the mean differences in FVC absolute values from baseline increase by 0.2 liter (5.6%, *n* = 36) over 3 months and decreased by 0.004 liter (3%, *n* = 26) over 12 months. Consistent with their findings, our study also demonstrated the substantial utility of early physiological changes in predicting the prognosis of patients undergoing pirfenidone treatment for IPF. Our data analysis further strengthens the growing body of evidence emphasizing the significance of monitoring and assessing physiological parameters at an early stage to provide valuable prognostic insights for IPF patients. These findings underscore the potential of early physiological assessments as valuable predictive tools, enabling healthcare providers to make informed treatment decisions and optimize patient management strategies.

Data obtained from clinical trials (13, 14) consistently indicate a notable association between the extent of FVC decline and the deterioration of health-related quality of life (HRQoL) as assessed through patient-reported outcomes measures like the St George’s Respiratory Questionnaire (SGRQ). These findings emphasize that a more substantial worsening of FVC aligns with a greater decline in HRQoL, underscoring the interdependency between pulmonary function and the subjective well-being reported by patients. By utilizing patient-reported outcome measures such as the SGRQ, clinicians and researchers gain valuable insights into the impact of FVC decline on the overall quality of life experienced by individuals with respiratory conditions. In our study, HRQoL measured with the SGRQ improved over time (lower score), and this improvement was maximal between 3 and 6 months of therapy (Mean SGRQ from −5.82 to −10.44, *p* = 0.02) in patients who continued to receive pirfenidone. We also recorded CAT as another tool to evaluate health-related quality of life. Results indicated CAT improvement after 3 months of pirfenidone therapy and persistent effect over 12 months (Mean CAT score from −2.5 to −6.08, *p* = 0.001). Additionally, it is important to note that the connection between the decline in FVC and HRQoL assessed by instruments such as the SGRQ or the CAT in patients with IPF is unlikely to follow a linear pattern. Instead, the impact of the same absolute reduction in FVC on HRQoL tends to be more pronounced as the disease progresses, especially when patients have already experienced substantial loss of lung volume and compromised gas exchange capacity, resulting in diminished physiological reserve. This implies that even modest declines in FVC during later stages of the disease can have a significant and potentially profound effect on the overall quality of life reported by patients. The non-linear relationship between FVC decline and HRQoL underscores the need for tailored treatment approaches that consider disease progression and the individual circumstances of IPF patients to optimize their well-being and maintain an acceptable quality of life.

Clinical evidence has substantiated the efficacy of pirfenidone in slowing down the progression of IPF and potentially even mitigating the occurrence of acute exacerbations (AE). When considering the risk factors associated with AE in IPF patients, it has been observed that AE tends to be more prevalent among individuals who have reached advanced stages of physiological and functional decline. These findings highlight the importance of identifying patients with IPF who are at a more advanced disease stage, as they may be particularly susceptible to experiencing acute exacerbations. By recognizing and closely monitoring this high-risk subgroup, healthcare professionals can implement proactive measures to prevent or manage exacerbations effectively, ultimately improving patient outcomes and enhancing their overall quality of life (15). In our study, AE rate was 22% (*n* = 11) within 12 months, and the average time to AE was 188 ± 75 days. In recent clinical trials, cohort studies on IPF, especially in Asian countries, generally reported a higher incidence of AE, and death caused by AE was somewhat higher in a survey of Japanese patients with IPF (16, 17). Another recent study in Japan also showed a high AE rate of approximately 26% with pirfenidone use (18).

In real-world settings, the tolerability of pirfenidone was evaluated, revealing that approximately 20.9% of patients with IPF discontinued the drug due to adverse events. This highlights the importance of closely monitoring and managing the potential side effects associated with pirfenidone treatment in clinical practice. While pirfenidone has demonstrated efficacy in IPF management, healthcare providers should carefully assess individual patient tolerability and closely monitor any adverse events to ensure optimal treatment outcomes (19). Gastrointestinal and skin-related adverse reactions were the most frequent events that led to pirfenidone discontinuation in these reports (8). Similar results were also noted in our study, with 32% gastrointestinal and 28% dermatologic adverse events. Side effects including gastrointestinal toxicity (19%) and skin toxicity (14%) were most likely to occur when taking pirfenidone for 3 months. However, a low discontinuation rate (4%, *n* = 2) and dose adjustment (10%, *n* = 5) were observed in our study. In the CAPACITY (Studies 004 and 006) and ASCEND trials, treatment was discontinued because of AEs occurred in 15% and 14.4% of patients in the pooled pirfenidone groups, respectively. The results showed that pirfenidone has a good tolerance to side effects in real-world practice in Taiwan. Significantly, dose adjustments implemented to address side effects during clinical trials did not diminish the therapeutic benefits of pirfenidone in slowing the decline of lung function. These findings underscore the importance of individualized treatment approaches and close monitoring to maximize the benefits of pirfenidone while minimizing potential side effects for patients with IPF (20).

Upon combining the ASCEND and CAPACITY study populations, the use of pirfenidone demonstrated a notable reduction in overall all-cause mortality. Specifically, at the 52 weeks mark, the incidence of all-cause mortality was observed to be 3.5% in the group receiving pirfenidone (2,403 mg/day), while the placebo group exhibited a higher rate of 6.7%. This finding highlights the potential life-saving benefits associated with pirfenidone treatment in patients with the condition under study. By reducing the risk of all-cause mortality, pirfenidone offers a promising therapeutic approach for individuals afflicted with the respective disease, ultimately improving their chances of survival and overall prognosis (7). However, in a real-world study conducted in South Korea, the 1 year all-cause mortality rate was 12.1% (21). In this study, the all-cause mortality rate was 16%. This may be related to the high GAP score and low BMI (24.20 ± 4.04) in our study group. In a study conducted by Fang et al. (22), it was observed that patients with a body mass index (BMI) below 25 kg/m^2^ faced a greater risk of disease progression, acute exacerbation, and mortality compared to overweight patients with a BMI above 25 kg/m^2^. These findings suggest that maintaining a higher BMI may confer some protective effects and potentially serve as a prognostic factor in the context of disease progression and outcomes. Identifying the relationship between BMI and disease severity can aid in risk stratification and the development of personalized treatment plans for patients. However, further research is needed to elucidate the underlying mechanisms behind these associations and determine the optimal BMI range for better outcomes in the specific population studied.

After 1 year of administering Pirfenidone, the research has detected favorable outcomes. Specifically, it has demonstrated a noticeable increase in progression-free survival and a reduction in the decline of FVC by more than 10%, or a reduction in mortality by 43.8%. Nonetheless, another study has indicated that FVC improvement was observed in only a limited subset of patients who were prescribed Pirfenidone. However, it is worth noting that in over 50% of cases, the use of Pirfenidone for 3 years managed to stave off a decrease in FVC (23, 24). The clinical effectiveness in mitigating the decline of FVC was particularly notable in patients with an initial FVC measurement of less than or equal to 75% of the predicted value or those who had experienced a decline of 150 milliliters or more in FVC over the 6 months preceding their initiation of Pirfenidone treatment (25, 26). The side effects occurred most in 6 months with skin lesion (25%) and GI upset (17.5%), especially in higher dose groups (1800 mg/day) (5, 27). Pirfenidone does indeed exhibit GI adverse effects; however, these can be mitigated through patient education and the guidance of healthcare professionals. It’s noteworthy that approximately 84% of individual’s experience anorexia as a side effect, which can be ameliorated by reducing the dosage. It’s also worth mentioning that these adverse effects tend to be more pronounced in patients who are concurrently taking oral steroids or N-acetylcysteine (26, 28, 29).

IPF often comes hand-in-hand with various comorbidities, notably lung cancer. Interestingly, pirfenidone could potentially prove efficacious in treating other medical conditions as well. While our particular case did not involve lung cancer, an intriguing study has surfaced. It suggests that among IPF patients who were concurrently prescribed antifibrotic medication, there was a noteworthy reduction of 39% in all-cause mortality (with a hazard ratio of 0.61 and a *p*-value of 0.006) when compared to those who were not administered antifibrotic medication, despite the presence of lung cancer (30). Cardiac function is typically not routinely assessed in patients with pulmonary fibrosis because the main focus of care for these individual’s centers on their lung condition. Pulmonary fibrosis primarily involves lung tissue scarring and directly affects breathing. Although heart issues can occasionally emerge as a secondary concern, they are not the primary focus of attention (31). Pirfenidone could potentially offer diverse therapeutic benefits in immune-mediated conditions like psoriasis, all while minimizing the immunosuppressive side effects commonly associated with current antipsoriatic medications (32). However, our case collection did not include patients with psoriasis, so we were unable to observe relevant phenomena.

The present study had several limitations that should be considered when interpreting the longitudinal outcomes of this analysis. First, it was a single-arm, prospective observational study with a small sample size. Prior to conducting the study, a sample size calculation was not carried out with respect to the primary outcome. Additionally, it is important to acknowledge that the study population exhibited a certain degree of heterogeneity among the different groups. These factors should be taken into consideration when interpreting the results and drawing conclusions from the study findings. While the absence of a sample size calculation may limit the statistical power of the study, the non-homogeneous nature of the study population can introduce variability and potential confounding factors that should be carefully addressed in the analysis and interpretation of the data. We could not analyze the effects of baseline characteristics, including age, sex, comorbidity, medication, and others. According to the most recent international treatment guidelines, the use of anti-acid medications in patients with IPF and asymptomatic gastroesophageal reflux disease (GERD) is conditionally recommended. This implies that while there is some evidence supporting the use of these medications in such cases, there may also be factors that warrant individualized decision-making and considerations. The conditional recommendation recognizes the potential benefits of anti-acid medications in managing GERD-related symptoms and their potential impact on IPF progression. However, it also emphasizes the need for careful evaluation of each patient’s specific clinical situation, taking into account their overall health, risk factors, and potential interactions with other treatments. It is crucial for healthcare providers to weigh the potential benefits and risks on a case-by-case basis, engaging in shared decision-making with patients to determine the most appropriate course of treatment for their specific needs (3). Nevertheless, it is important to note that the current recommendation for the use of anti-acid medications in the treatment of IPF lacks supportive evidence from randomized controlled trials. Consequently, the efficacy and potential benefits of these medications in IPF management continue to be a topic of ongoing debate. The absence of robust clinical trial data underscores the need for further research to elucidate the true value and potential impact of anti-acid medications in IPF treatment. As the scientific community continues to explore and investigate the role of these medications, it is essential for healthcare providers to exercise caution and consider the individualized needs and preferences of patients when making treatment decisions. Collaborative discussions between healthcare professionals and patients are vital to ensure that the most appropriate and evidence-based approaches are employed in the management of IPF (33). Moreover, other therapies commonly used in the treatment of IPF (e.g., N-acetylcysteine, steroids, bronchodilators) have not been effective in slowing the progression of IPF (34). Second, under the payment regulations of Taiwan National Health Insurance, the continuous supply of pirfenidone must be established so that lung function (FVC) cannot be decreased by more than 10% within 6 months. In the real world, the number of patients lost over time is due to the discontinuous supply of pirfenidone and death. This will lead to a selective bias of the survivor effect and even improve the pulmonary function test. It is important to acknowledge that the observed physiological changes following pirfenidone therapy in this study may not necessarily indicate a direct therapeutic effect. Additionally, due to the nature of being an observational study, there is a possibility of potential omissions in obtaining pulmonary function tests at irregular intervals. These factors should be taken into consideration when interpreting the study findings and drawing conclusions. While the observed physiological changes may provide valuable insights, further research, including randomized controlled trials, is warranted to establish a more conclusive understanding of the therapeutic effects of pirfenidone in the specific context studied. Implementing rigorous study designs and standardized monitoring protocols can help mitigate potential limitations and enhance the validity and reliability of the results obtained.

## Conclusion

Our real-world study describe, for the first time in Taiwan, the use of pirfenidone over a 12 months period. It preserves the lung function and improves quality of life with tolerable side effects. The most common side effects of pirfenidone include gastrointestinal issues and photosensitivity, which are typically managed by reducing the dosage. Overall, pirfenidone is an effective and well-tolerated anti-fibrotic medication that provides a practical real-world treatment option for individuals with IPF. Nonetheless, further research is needed to unravel the underlying mechanisms behind these early physiological changes and their precise implications for IPF prognosis. A deeper understanding of the intricate relationship between pirfenidone, disease progression, and physiological response will empower healthcare professionals to refine treatment strategies and improve patient outcomes.

## Data availability statement

The raw data supporting the conclusions of this article will be made available by the authors, without undue reservation.

## Ethics statement

Written informed consent was obtained from the individual(s) for the publication of any potentially identifiable images or data included in this article.

## Author contributions

Che-YC and S-CC contributed in data collection and analysis. Y-FW, Chu-YC, and Y-CL helped finalize study concept and study design. P-WH, J-CH, and C-HC contributed to the data collection and analysis. Che-YC, S-CC, and Y-CL drafted the manuscript. All authors contributed to the article and approved the submitted version.

## Figures and Tables

**Table:** 

IPF	Idiopathic pulmonary fibrosis
TGF-β	Transforming growth factor beta
TNF-ɑ	Tumor necrosis factor alpha
VC	Vital capacity
PFS	Progression free survival
FVC	Forced vital capacity
EMA	European Medicines Agency
FDA	Food and Drug Administration
NHI	National Health Insurance
HRCT	High resolution chest tomography
SGRQ	St. George’s respiratory questionnaire
COPD	Chronic obstructive pulmonary disease
CAT	Chronic obstructive pulmonary disease assessment test
IRB	Institutional Review Board
mMRC	Modified Medical Research Council
QOL	Quality of life
DLCO	Diffusing capacity for carbon monoxide
AE	Acute exacerbation
GERD	Gastroesophageal reflux disease
