# Supplementary material for: Real world experience on the effectiveness and safety of pirfenidone in patients with idiopathic pulmonary fibrosis in Taiwan
Source: Front Med (Lausanne). 2023 Oct 30;10:1242260. doi: 10.3389/fmed.2023.1242260 (PMC10642852; doi:10.3389/fmed.2023.1242260)
Supplement: Supplementary file 1 [file Data_Sheet_1.docx]

Supplement Fig 1. Different methods for imputing missing data of predicted FVC (%)

| **Complete cases** | **Last Observation Carried Forward** |
| --- | --- |
|  |  |
| **Multiple Imputation** |  |
|  |  |

Supplement table 1. Sensitivity analysis for imputation methods of missing data in predicted FVC (%)

| Method | Complete cases | LOCF | MI |
| --- | --- | --- | --- |
| Number of Patients | 16 | 50 | 50 |
| P value | 0.151 | 0.059 | 0.173 |

P value: Friedman test

LOCF: Last Observation Carried Forward

MI: Multiple Imputation

Supplement Fig 2. Different methods for imputing missing data of FVC (L)

| **Complete cases** | **Last Observation Carried Forward** |
| --- | --- |
|  |  |
| **Multiple Imputation** |  |
|  |  |

Supplement table 2. Sensitivity analysis for imputation methods of missing data in FVC (L)

| Method | Complete cases | LOCF | MI |
| --- | --- | --- | --- |
| Number of Patients | 16 | 50 | 50 |
| P value | 0.200 | 0.055 | 0.001^a^ |

P value: Friedman test

LOCF: Last Observation Carried Forward

MI: Multiple Imputation

a: Bonferroni adjustment, M6 = M3 > Baseline = M9 = M12. “=” indicates p value >0.05, otherwise, p value <0.05.

Supplement Fig 3. Different methods for imputing missing data of predicted DLCO (%)

| **Complete cases** | **Last Observation Carried Forward** |
| --- | --- |
|  |  |
| **Multiple Imputation** |  |
|  |  |

Supplement table 3. Sensitivity analysis for imputation methods of missing data in predicted DLCO (%)

| Method | Complete cases | LOCF | MI |
| --- | --- | --- | --- |
| Number of Patients | 6 | 29 | 50 |
| P value | 0.051 | 0.002^a^ | <0.001^b^ |

P value: Friedman test

LOCF: Last Observation Carried Forward

MI: Multiple Imputation

a: Bonferroni adjustment, M12 < Baseline = M3 = M9 = M12. “=” indicates p value >0.05, otherwise, p value <0.05.

b: Bonferroni adjustment, M6=M12 < Baseline = M3 = M9. “=” indicates p value >0.05, otherwise, p value <0.05.

Supplement Fig 4. Different methods for imputing missing data of SGRQ

| **Complete cases** | **Last Observation Carried Forward** |
| --- | --- |
|  |  |
| **Multiple Imputation** |  |
|  |  |

Supplement table 4. Sensitivity analysis for imputation methods of missing data in SGRQ

| Method | Complete cases | LOCF | MI |
| --- | --- | --- | --- |
| Number of Patients | 24 | 38 | 50 |
| P value | 0.007^a^ | 0.002^a^ | <0.001^a^ |

P value: Friedman test

LOCF: Last Observation Carried Forward

MI: Multiple Imputation

a: Bonferroni adjustment, M3 = M6 = M9 =M12 < Baseline. “=” indicates p value >0.05, otherwise, p value <0.05.

Supplement Fig 5. Different methods for imputing missing data of CAT

| **Complete cases** | **Last Observation Carried Forward** |
| --- | --- |
|  |  |
| **Multiple Imputation** |  |
|  |  |

Supplement table 5. Sensitivity analysis for imputation methods of missing data in CAT

| Method | Complete cases | LOCF | MI |
| --- | --- | --- | --- |
| Number of Patients | 24 | 38 | 50 |
| P value | <0.001^a^ | <0.001^a^ | <0.001^a^ |

P value: Friedman test

LOCF: Last Observation Carried Forward

MI: Multiple Imputation

a: Bonferroni adjustment, M6 = M9 =M12 < M3 = Baseline. “=” indicates p value >0.05, otherwise, p value <0.05.
